# RES1/384: Spreading the Use of Kinetic Modeling Techniques by JAVA Analysis Software

**DOI:** 10.2196/jmir.1.suppl1.e77

**Published:** 1999-09-19

**Authors:** P Rudnicki, K Mikolajczyk, M Grodzki, C Burger

**Affiliations:** ^1^Warsaw University of TechnologyWarsawPoland and University HospitalZurichSwitzerland

**Keywords:** Medical Informatics Applications, PET, Kinetic Modeling, Java

## Abstract

**Introduction:**

Kinetic modeling is the method of choice for assessing the behaviour of new PET (Positron Emission Tomography) tracers. For suitable tracers, kinetic models allow to derive unique functional information from the acquired PET data, for instance the absolute perfusion or the density of specific receptors in brain tissue. However, the processing steps required are sophisticated. As there has no comprehensive modeling software been available in the past, kinetic models could only be developed and applied by a limited number of sites. This paper presents such a software package called PMOD. Being developed with Internet technologies it can easily be distributed and may thus help consolidate more widespread use of kinetic modeling.

**Methods:**

Aiming at maximal portability, the entire software was programmed in Java 2. An interface was defined such that new models can easily and seamlessly be added by a sort of plug-in programming. It is general enough to cope with virtually all models published so far. Innovative models may therefore directly be implemented in PMOD, or they may easily be incorporated even by external researchers. The supported features include weighted least squares fitting, parameter coupling among models, Monte Carlo simulations for assessing parameter identifiability, and batch processing for scheduling a sequence of time-consuming trials. The software can be configured as a local JAVA application, but can also be installed on an Internet server and be run from any Java2-enabled WWW-browser.

**Results:**

The modeling software currently supports 19 different models ranging from simple tissue ratio methods to complex multi-injection protocols with two input curves plus metabolite correction. It has been tested on different platforms such as HP-UX, Sun Solaris, Linux, and Windows. At present it has been adopted by 7 sites which run the software on PC/NT. Experiences on this platform demonstrate:

1. The Java Virtual Machine runs with high reliability.
2. Despite just-in-time compilation there still exists a significant performance penalty for Java applications, especially with respect to memory management.

The kinetic modeling environment in its present form is used on a daily basis at several sites for scientific studies and even for some types of clinical studies.

**Discussion:**

Earlier kinetic modeling programs were typically based on high-level languages such as Matlab or IDL and tailored to the need of individual sites. Every attempt to port them to a different environment was a major undertaking. This is in contrast to the present modeling software which runs on any platform as an application and supports easy data input.

Acknowledgement:

This work was supported by the Swiss National Science Foundation, Project 7PLPJ048289.

